# No Mid-Term Benefits of Glucagon-Like Peptide-1 (GLP-1) Receptor Agonists Following Total Joint Arthroplasty: A Systematic Review

**DOI:** 10.2106/JBJS.25.00879

**Published:** 2026-04-01

**Authors:** Filippo Leggieri, Simon N. van Laarhoven, Vicente J. León Muñoz, Joaquin Moya-Angeler, Mustafa Akkaya, Roberto Civinini, Matteo Innocenti

**Affiliations:** 1 Department of Clinical Orthopaedics, University of Florence, Florence, Italy; 2 Department of Orthopaedic Surgery, Sint Maartenskliniek, Nijmegen, The Netherlands; 3 Instituto de Cirugía Avanzada de la Rodilla (ICAR), Murcia, Spain; 4 Department of Orthopaedic Surgery and Traumatology, Hospital General Universitario Reina Sofía, Murcia, Spain; 5 Department of Orthopaedics & Traumatology, Yüksek İhtisas University, Ankara, Türkiye; 6 Department of Orthopaedics & Traumatology, Ankara Güven Hospital, Ankara, Türkiye

## Abstract

**Background:**

The aim of this systematic review was to evaluate the impact of glucagon-like peptide-1 receptor agonists (GLP-1 RAs) on medical complications, implant failure rates, and health-care-related costs in patients undergoing hip or knee arthroplasty.

**Methods:**

A comprehensive search of electronic databases, including PubMed, Embase, Web of Science, the Cochrane Library, the World Health Organization International Clinical Trials Registry Platform (ICTRP), and the UK Clinical Trials Gateway, was conducted and was limited to studies from database inception to March 31, 2025. Inclusion criteria comprised randomized controlled trials or cohort studies involving adults (≥18 years old) undergoing total joint arthroplasty (TJA) while receiving a GLP-1 RA treatment of any dosage or duration. The risk of bias was assessed using the Cochrane risk-of-bias tool and ROBINS-I (Risk Of Bias In Non-Randomized Studies - of Interventions) assessment. Due to substantial heterogeneity in the study designs, a qualitative synthesis approach was employed.

**Results:**

Eight retrospective studies met the inclusion criteria, encompassing 22,611 GLP-1 RA users and 77,810 controls. The mean patient age ranged from 56 to 64 years. Hospital readmission rates showed the most consistently favorable results among GLP-1 RA users, with 3 studies reporting significant reductions of 29% to 47% during the 90-day postoperative period. Five studies demonstrated that GLP-1 RA use was associated with significant reductions, ranging from 30% to 44%, in periprosthetic joint infection (PJI) rates, whereas 3 studies found no significant differences. Hospital resource utilization favored GLP-1 RA therapy, with several studies demonstrating shorter hospital stays and lower 90-day costs. Medical complications yielded variable results: some studies reported increased vascular and pulmonary events among GLP-1 RA users, whereas others observed reduced sepsis and hypoglycemic events in those patients.

**Conclusions:**

GLP-1 RA therapy was associated with reduced hospital readmissions and decreased hospital costs within 90 days postoperatively, although its benefits for PJI prevention showed mixed results, with some studies demonstrating significant reductions in PJI while others showed no difference. No consistent clinical advantages were observed at the 2-year follow-up.

**Level of Evidence:**

Therapeutic Level III. See Instructions for Authors for a complete description of levels of evidence.

Patient obesity represents a major concern in total hip and knee arthroplasty because it has been shown to adversely impact surgical procedures, perioperative management, rehabilitation protocols, and clinical outcomes^1-6^. Effective glycemic control is also essential for optimal surgical outcomes, as perioperative hyperglycemia has been strongly associated with higher risks of complications^7,8^, including surgical site infections, delayed wound healing, and a prolonged hospital stay^9,10^. Despite its association with complications, obesity has not been shown to significantly affect postoperative functional outcomes or patient satisfaction^11,12^.

Glucagon-like peptide-1 receptor agonists (GLP-1 RAs) have gained attention in arthroplasty care for their potential benefits, such as enhanced satiety^13-15^, beneficial effects on insulin regulation^16,17^, and cardiovascular protective effects, including reduced rates of myocardial infarction, stroke, and cardiovascular mortality^18-20^. Due to these diverse pharmacodynamics that encompass metabolic, cardiovascular, and weight-management benefits, GLP-1 RA has been an increasingly popular option for comprehensive patient optimization^21,22^. Beyond immediate weight reduction, advantages in the long term may include reduced late-onset periprosthetic joint infection (PJI), improved implant survivorship, and enhanced quality of life through the synergistic effects of sustained weight loss and osteoarthritis resolution.

Although recent reviews have suggested potential benefits of GLP-1 RAs in arthroplasty outcomes, these reviews had methodological limitations, including the inappropriate pooling of heterogeneous studies^23^ and the mixing of preclinical and clinical data^24^. A rigorous qualitative synthesis is therefore warranted. The aim of the present systematic review was to consolidate the current evidence on medical complications, implant failure rates, and hospital costs in patients undergoing total hip arthroplasty (THA) or total knee arthroplasty (TKA) while receiving GLP-1 RAs.

## Materials and Methods

This systematic review adhered to the Preferred Reporting Items for Systematic Reviews and Meta-Analyses (PRISMA) guidelines^25^. The review was not registered on PROSPERO, and the study protocol was not published prior to submission. A comprehensive search of electronic databases, including PubMed, Embase, Web of Science, the Cochrane Library, the World Health Organization International Clinical Trials Registry Platform (ICTRP), and the UK Clinical Trials Gateway, was performed to identify relevant studies. The search was limited to articles from database inception (i.e., the earliest available records) to March 31, 2025. The combination of search terms used to systematically retrieve pertinent studies can be found in the Appendix.

### Eligibility Criteria

Articles were filtered using Population, Intervention, Comparison, Outcomes, and Study design (PICOS) criteria (Table 1). The inclusion criteria were published or unpublished randomized controlled trials or cohort studies in adults (≥18 years old) undergoing TKA or THA at any disease stage while receiving a GLP-1 RA treatment of any dosage or duration. The exclusion criteria were inaccessible full texts and crossover trials.

**Table 1:** PICOS Question

Population	Adult patients undergoing hip or knee arthroplasty
Intervention	Weight-loss GLP-1 RA medications
Comparison	No weight-loss medications
Outcomes	Medical complication
	Implant failure
	All-time implant failure
	All-time revision surgery
Study	Randomized controlled trials or cohort studies

### Study Selection

**Fig. 1:**
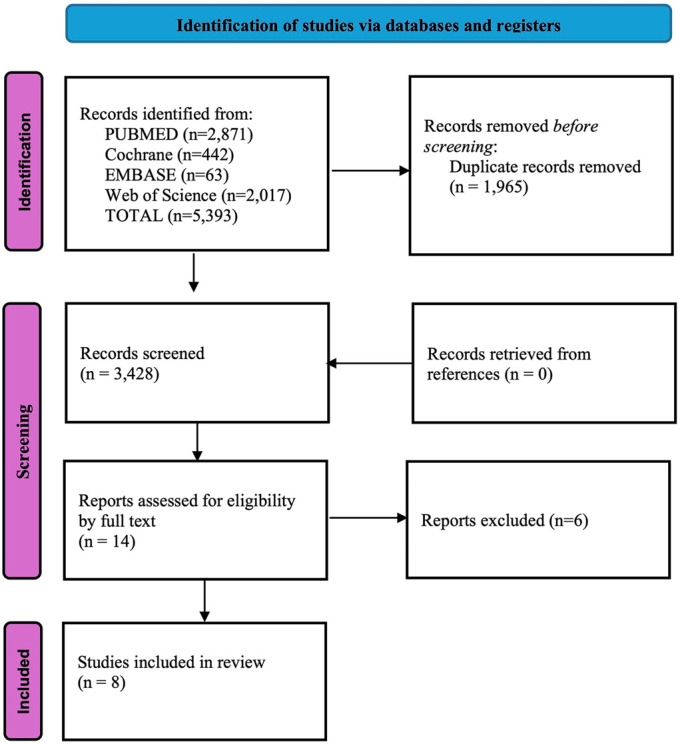
PRISMA flowchart of the included studies.

Zotero (version 6.0.37; Corporation for Digital Scholarship [2023]) was used to remove duplicates. Two independent reviewers screened titles and abstracts, then acquired eligible full texts. The citation sections of the selected articles were examined for additional relevant literature. Disagreements between reviewers were resolved by a senior author. The first unmet criterion was recorded as the primary reason for exclusion; a detailed list of the excluded studies is provided in the Results. The selection process is summarized in the PRISMA flowchart (Fig. 1).

### Data Extraction

Two independent reviewers extracted data including first author, title, publication year, study design, sample size, surgery type (THA or TKA), follow-up duration, GLP-1 RA protocol, and the rates of surgical site infection, PJI, revision surgery, and medical complications at 90 days, 1 year, and 2 years. Hospital resource utilization data were collected when available. Revision arthroplasty was defined as any operation exchanging femoral, tibial, or mobile components following TKA, or exchanging femoral, acetabular, or mobile components following THA. Data were entered electronically by 1 reviewer and verified by another.

### Risk-of-Bias Assessment

Two review authors independently assessed the risk of bias for each included study with use of the Cochrane risk-of-bias tool, following the guidelines outlined in the Cochrane Handbook for Systematic Reviews of Interventions. Additionally, each study was assigned a quality rating with use of the ROBINS-I (Risk Of Bias In Non-Randomized Studies - of Interventions) risk assessment tool^26^. Disagreements were resolved by discussion or by deferring to a third review author. When information was missing from the published papers, the study authors were contacted. The detailed risk-of-bias assessments and judgments for each study are provided in the Appendix.

### Statistical Analysis

Due to substantial heterogeneity in the study designs, populations, interventions, and reported outcomes, a qualitative synthesis approach was employed. Descriptive statistics were used to summarize study characteristics, and risk ratios (RRs), odds ratios (ORs), or hazard ratios (HRs), with 95% confidence intervals (CIs), were extracted when available. Significance was defined as p < 0.05.

## Results

Upon evaluation of the full texts, 6 articles were excluded on the basis of the following criteria: 1 study was eliminated due to combined data of GLP-1 RA with other medications^13^, 1 study was excluded for being a transcription of an oral abstract^27^, 3 studies were excluded because they evaluated patients prior to hip or knee surgery rather than postoperatively^28-30^, and 1 study was excluded because it assessed non-orthopaedic patients^31^.

### Study Characteristics

Eight studies examined GLP-1 RA use in patients undergoing primary THA or TKA, encompassing 22,611 GLP-1 RA users and 77,810 controls^32-39^ (Tables 2 and 3). Only primary THA and TKA cases were included. The mean patient age ranged from 56 to 64 years. Most studies reported a mean age of 60 to 64 years for both groups^32,35-38^, while 2 studies reported that the majority of the patients were in the range of 55 to 69 years of age^33,34^. TKA studies showed greater female representation (60% to 67% of patients) than THA studies (42% to 69% of patients)^32,35,36,38^. The prevalence of diabetes (37% to 100%) varied considerably on the basis of the inclusion criteria, with some studies targeting only diabetic populations^33,34,37^. After propensity matching, comorbidity indices were well-matched between the groups, including rates of obesity (or obesity/overweight), insulin use, and metformin use^33-35,39^. However, despite propensity matching, 1 study^38^ reported substantially higher rates of insulin use (65% versus 36%) and metformin use (68% versus 43%) in the GLP-1 RA group compared with the control group.

**Table 2:** Design Characteristics of the Included Studies*

Study	Year	Design	Sample Size	Type of Surgery	Follow-up	GLP-1 RA Protocol
Katzman et al.^38^	2025	Retrospective, PS matching	865 GLP-1 RA users matched with 8,650 non-users	TKA	Mean, 2.2 years (GLP-1 RA users) vs. 2.9 years (non-users)	6 months preop. and continued up to 3 months postop.
Buddhiraju et al.^35^	2024	Retrospective, PS matching	THA: 1,044 GLP-1 RA users matched with 1,044 non-users; TKA: 2,095 GLP-1 RA users matched with 2,095 non-users	THA and TKA	90 days	Between 1 year and 15 days preop.
Magruder et al.^34^	2023	Retrospective, PS matching	7,051 semaglutide users matched with 34,524 non-users	TKA	90 days and 2 years	Active semaglutide prescription at time of TKA
Verhey et al.^39^	2025	Retrospective, PS matching	5,345 GLP-1 RA users matched with 5,345 non-users	THA	90 days and 2 years	GLP-1 RA therapy at time of THA
Heo et al.^37^	2025	Retrospective cohorts	812 GLP-1 RA users, 3,248 non-users	THA	90 days and 1 year	GLP-1 RA use (at least 3 fills within 6 months preop. or 1 fill of ≥90-day supply within 6 months preop.)
Magruder et al.^33^	2024	Retrospective, PS matching	1,653 semaglutide users, 7,812 non-users	THA	90 days and 2 years	Semaglutide use at time of THA
Kim et al.^36^	2025	Retrospective, 3 cohorts	Severe obesity (no GLP-1): 5,949; morbid obesity + GLP-1: 2,975; morbid obesity + no GLP-1: 2,975	TKA	90 days and 2 years	3 months preop. and postop.
Kim et al.^32^	2025	Retrospective, 3 cohorts	Severe obesity (no GLP-1): 3,084; morbid obesity + GLP-1: 771; morbid obesity + no GLP-1: 3,084	THA	90 days and 2 years	3 months preop. and postop.

* PS = propensity score.

**Table 3:** Patient Baseline Characteristics in the Included Studies*

Study	Surgery	No. of Patients	Mean Age *(yr)*	% Women	BMI *(kg/m*^*2*^*)*	Diabetes Prevalence *(%)*	Key Comorbidities	Notable Outcomes
Katzman et al.^38^	TKA	GLP-1: 865; control: 8,650	64 in both groups	GLP-1: 66.0%; control: 66.3%	1 yr preop.: GLP-1, 36.0; control, 35.7. Day of surgery: GLP-1, 35.9; control, 36.1	GLP-1: 74.5%; control: 37.5%	• Long-term insulin use: GLP-1, 65.1%; control, 35.5% • Metformin use: GLP-1, 68.3%; control, 42.7% • Higher CCI in GLP-1 (4.4 vs. 3.2)	BMI difference between groups diminished by the day of surgery and remained similar postop.
Buddhiraju et al.^35^	THA	GLP-1: 1,044; control: 1,044	GLP-1: 63.3; control: 63.5	GLP-1: 51.1%; control: 52.0%	Not reported	GLP-1: 69.3%; control: 68.3%	• Obesity/overweight: GLP-1, 70.3%; control, 73.2% • Hypertension: GLP-1, 80.3%; control, 81.7% • HbA1c ≥7.5%: GLP-1, 41.7%; control, 39.8%	Similar HbA1c levels between groups
Buddhiraju et al.^35^	TKA	GLP-1: 2,095; control: 2,095	GLP-1: 64.1; control: 64.2	GLP-1: 60.7%; control: 60.9%	Not reported	GLP-1: 68.7%; control: 68.4%	• Obesity/overweight: GLP-1, 68.8%; control, 70.6% • Hypertension: GLP-1, 78%; control, 80.6% (p = 0.039) • HbA1c ≥7.5%: GLP-1, 37.2%; control, 33.7% (p = 0.01)	Lower infection, aspiration, and DVT rates in GLP-1 group (not significant)
Magruder et al.^34^	TKA	Semaglutide: 7,051; control: 34,524	Majority 55-69 in both groups†	Semaglutide: 61.4%; control: 61.6%	Not reported	Complicated DM: semaglutide, 44.5%; control, 44.2%	• Obesity: semaglutide, 86.2%; control, 86.4% • Insulin use: semaglutide, 55.0%; control, 54.7% • Metformin use: semaglutide, 90.1%; control, 90.4%	Closely matched cohorts
Verhey et al.^39^	THA	GLP-1: 5,345; control: 5,345	GLP-1: 57; control: 57	After matching: GLP-1, 69%; control, 69%	Not reported	Not directly reported	After matching (comparable between groups): • Obesity: GLP-1, 68.2%; control, 68.3% • Depression: GLP-1, 33.4%; control, 33.4% • Tobacco use: GLP-1, 29.9%; control, 29.8%	Significant differences in demographics before matching; well-balanced after matching
Heo et al.^37^	THA	GLP-1: 812; control: 3,248	GLP-1: 61; control: 60	GLP-1: 41.7%; control: 42.4%	Not reported	100% (T2DM study)	• Insulin-dependent DM: GLP-1, 20.8%; control, 17.6% • Complicated diabetes: GLP-1, 49.4%; control, 47.5% • CHF: GLP-1, 12.4%; control, 10.9%	No significant differences between groups
Magruder et al.^33^	THA	Semaglutide: 1,653; control: 7,812	Majority 55-69 in both groups†	Semaglutide: 47.6%; control: 47.8%	Not reported	Complicated T2DM: semaglutide, 39.7%; control, 39.4%	• Obesity: semaglutide, 84.9%; control, 85.2% • Insulin use: semaglutide, 47.3%; control, 46.2% • Metformin use: semaglutide, 93.2%; control: 93.6%	Well-matched cohorts; hospital LOS not reported
Kim et al.^36^	TKA	Severe obesity: 5,949; morbid obesity + GLP-1: 2,975; morbid obesity + no GLP-1 (control): 2,975	∼62.2 in each group	∼66.8% across all groups	Severe obesity: 35-39.9; morbid obesity: ≥40	∼52.8% across all groups	• CCI: ∼3.3 across all groups • Smoking: lower rate in GLP-1 group compared with the severe obesity and morbid obesity control groups (46.6% vs. 50.0% and 49.7%, respectively) • Alcohol abuse: lower in GLP-1 group (5.0% vs. 7.7% vs. 8.0%)	GLP-1 group had a shorter hospital LOS (2.7 vs. 2.7 vs. 2.9 days; p = 0.047)
Kim et al.^32^	THA	Severe obesity: 3,084; morbid obesity + GLP-1: 771; morbid obesity + no GLP-1 (control): 3,084	62.1 across all groups	GLP-1: 52.8%; control: 52.9%; severe obesity: 51.9%	Severe obesity: 35-39.9; morbid obesity: ≥40	∼52.3% across all groups	CCI: ∼3.3-3.4 across all groups	Significantly shorter hospital LOS in GLP-1 group (2.2 vs. 2.7 vs. 3.1 days; p = 0.001)

* CCI = Charlson Comorbidity Index, HbA1c = hemoglobin A1c, DVT = deep vein thrombosis, DM = diabetes mellitus, T2DM = type-2 diabetes mellitus, CHF = congestive heart failure, LOS = length of stay.

† Mean age was not reported in the primary study; the values represent the age range containing the majority of the patients.

### Study Designs

Five studies employed propensity score matching^33-35,38,39^. Uniquely, Kim et al.^32,36^ utilized a 3-cohort design, comparing (1) morbidly obese patients (body mass index [BMI], ≥40 kg/m^2^) who were using GLP-1 RA, (2) morbidly obese non-users, and (3) patients with severe obesity (BMI, 35 to 39.9 kg/m^2^) who were not using GLP-1 RA. Heo et al.^37^ specifically targeted diabetic patients undergoing THA, comparing outcomes without propensity matching.

### Surgical Complications (Table 4)

Five of 8 studies reported significant reductions in PJI associated with GLP-1 RA use. Buddhiraju et al.^35^ found reduced 90-day PJI among GLP-1 RA users (RR, 0.58; 95% CI, 0.34 to 0.99; p = 0.042), and both studies by Magruder et al. demonstrated reduced 2-year PJI (30%^33^ and 44%^34^ lower odds; p < 0.001 and p = 0.005, respectively). In their 3-cohort analyses, Kim et al. found lower 90-day PJI rates among GLP-1 RA users compared with both severely obese patients and morbidly obese non-users (TKA^36^: 1.0% versus 1.4% versus 1.8%, respectively [p = 0.028]; THA^32^: 1.6% versus 2.2% versus 3.2%, respectively [p = 0.01; OR, 0.47]). However, these differences disappeared at the 2-year follow-up in both cohorts^32,36^. The remaining 3 studies^37-39^ found no significant differences in PJI rates between GLP-1 RA users and controls. Revision surgery rates were significantly lower among GLP-1 RA users compared with controls in 3 studies: both of the Magruder et al. cohorts (TKA^34^: OR, 0.86 [p = 0.02]; THA^33^: OR, 0.64 [p = 0.0257]) and Katzman et al.^38^ (p = 0.034).

**Table 4:** Surgical Outcomes, Medical Complications, and Hospital Costs Reported in the Included Studies*

Outcome	Study	GLP-1 RA Group	Control Group	Effect Size (95% CI)	P Value	Significant†
SSI						
90-day SSI	Buddhiraju^35^ (THA)	1.00%	1.00%	RR, 1.01 (0.42-2.41)	0.988	No
90-day SSI	Verhey^39^	0.60%	0.40%	OR, 1.549 (0.905-2.652)	0.141	No
90-day SSI	Heo^37^	3.90%	5.20%	OR, 1.39 (0.94-2.06)	0.1	No
90-day SSI	Magruder^33^ (THA)	1.00%	1.40%	OR, 0.74 (0.43-1.21)	0.255	No
2-year SSI	Verhey^39^	0.6%	0.8%	OR, 1.471 (0.923-2.343)	0.129	No
PJI						
90-day PJI	Buddhiraju^35^ (THA)	2.10%	3.60%	RR, 0.58 (0.34-0.99)	0.042	Yes (↓)
90-day PJI	Verhey^39^	1.20%	1.10%	OR, 1.086 (0.761-1.550)	0.717	No
90-day PJI	Heo^37^	1.30%	1.20%	OR, 0.78 (0.39-1.55)	0.47	No
1-year PJI	Heo^37^	1.50%	1.40%	OR, 0.88 (0.46-1.68)	0.69	No
2-year PJI	Katzman^38^	1.20%	1.30%	Not specified	0.669	No
2-year PJI	Magruder^34^ (TKA)	2.10%	3.00%	OR, 0.70 (0.58-0.83)	<0.001	Yes (↓)
2-year PJI	Magruder^33^ (THA)	1.60%	2.90%	OR, 0.56 (0.37-0.82)	0.005	Yes (↓)
2-year PJI	Verhey^39^	2.7%	2%	OR, 1.168 (0.881-1.550)	0.314	No
Any-time PJI	Katzman^38^	1.20%	1.50%	Not specified	0.392	No
Revision surgery						
90-day revision	Buddhiraju^35^ (THA)	1.70%	2.80%	RR, 0.62 (0.34-1.13)	0.117	No
90-day revision	Buddhiraju^35^ (TKA)	0.60%	0.80%	RR, 0.72 (0.34-1.50)	0.375	No
90-day revision	Verhey^39^	0.70%	1.00%	OR, 0.783 (0.516-1.186)	0.292	No
1-year revision	Heo^37^	2.50%	2.70%	OR, 1.21 (0.71-2.00)	0.48	No
2-year revision	Verhey^39^	1.7%	1.7%	OR, 0.989 (0.738-1.325)	1	No
2-year revision	Magruder^34^ (TKA)	4.00%	4.50%	OR, 0.86 (0.75-0.98)	0.02	Yes (↓)
2-year revision	Magruder^33^ (THA)	1.80%	2.80%	OR, 0.64 (0.43-0.93)	0.0257	Yes (↓)
2-year all-cause revision	Katzman^38^	2.30%	2.60%	Not specified	0.362	No
Any-time revision	Katzman^38^	2.70%	3.90%	Not specified	0.034	Yes (↓)
90-day complications						
ED utilization	Buddhiraju^35^ (THA)	5.90%	6.60%	RR, 0.90 (0.55-1.47)	0.668	No
ED utilization	Buddhiraju^35^ (TKA)	7.20%	7.70%	RR, 0.93 (0.68-1.28)	0.66	No
ED visits	Verhey^39^	4.80%	5.80%	OR, 0.814 (0.686-0.965)	0.02	Yes (↓)
ED visits	Katzman^38^	5.90%	4.00%	Not specified	0.008	Yes (↑)
Readmission	Buddhiraju^35^ (TKA)	1.10%	2.00%	RR, 0.53 (0.31-0.90)	0.017	Yes (↓)
Readmission	Buddhiraju^35^ (THA)	1.60%	2.00%	RR, 0.81 (0.41-1.59)	0.532	No
Readmission	Verhey^39^	4.10%	4.50%	OR, 0.909 (0.754-1.096)	0.341	No
Readmission	Heo^37^	8.50%	8.70%	OR, 1.01 (0.76-1.34)	0.95	No
Readmission	Katzman^38^	4.30%	3.60%	Not specified	0.168	No
Readmission	Magruder^34^ (TKA)	7.00%	9.40%	OR, 0.71 (0.64-0.79)	<0.001	Yes (↓)
Readmission	Magruder^33^ (THA)	6.20%	8.80%	OR, 0.68 (0.54-0.84)	0.0004	Yes (↓)
Mortality	Verhey^39^	0.03%	0.10%	OR, 0.400 (0.178-2.061)	0.45	No
DVT	Buddhiraju^35^ (THA)	1.00%	1.10%	RR, 0.91 (0.39-2.13)	0.831	No
DVT	Verhey^39^	0.50%	0.60%	OR, 0.866 (0.512-1.467)	0.688	No
DVT	Heo^37^	1.00%	1.30%	OR, 1.21 (0.52-2.81)	0.65	No
DVT	Magruder^34^ (TKA)	0.80%	0.50%	OR, 1.50 (1.25-2.00)	0.007	Yes (↑)
DVT	Magruder^33^ (THA)	0%	0.70%	OR, 0.69 (0.30-1.38)	0.3348	No
PE	Buddhiraju^35^ (THA)	1.00%	1.00%	RR, 1.00 (0.42-2.38)	0.991	No
PE	Verhey^39^	0.10%	0.20%	OR, 0.889 (0.343-2.305)	1	No
PE	Magruder^34^ (TKA)	0.50%	0.60%	OR, 0.82 (0.56-1.15)	0.277	No
PE	Magruder^33^ (THA)	0%	0.40%	OR, 0.69 (0.24-1.62)	0.4453	No
VTE	Magruder^34^ (TKA)	1.10%	1.00%	OR, 1.10 (0.86-1.40)	0.41	No
VTE	Magruder^33^ (THA)	0.70%	0.90%	OR, 0.74 (0.37-1.35)	0.3601	No
CVA (stroke)	Magruder^34^ (TKA)	1.20%	0.90%	OR, 1.37 (1.07-1.74)	0.01	Yes (↑)
CVA	Magruder^33^ (THA)	0%	0.90%	OR, 0.67 (0.32-1.25)	0.2426	No
Acute renal failure	Buddhiraju^35^ (THA)	2.00%	1.40%	RR, 1.40 (0.65-3.00)	0.385	No
Acute renal failure	Buddhiraju^35^ (TKA)	2.20%	2.10%	RR, 1.05 (0.66-1.66)	0.853	No
MI	Magruder^34^ (TKA)	1.00%	0.70%	OR, 1.49 (1.13-1.94)	0.003	Yes (↑)
MI	Magruder^33^ (THA)	0%	0.70%	OR, 0.72 (0.33-1.39)	0.3579	No
PNA	Magruder^34^ (TKA)	2.80%	1.70%	OR, 1.67 (1.41-1.97)	<0.001	Yes (↑)
PNA	Magruder^33^ (THA)	1.90%	1.40%	OR, 1.37 (0.91-2.02)	0.1185	No
AKI	Verhey^39^	0.50%	0.60%	OR, 0.866 (0.512-1.466)	0.688	No
AKI	Heo^37^	4.30%	4.40%	OR, 0.99 (0.67-1.47)	0.96	No
AKI	Magruder^34^ (TKA)	4.90%	3.90%	OR, 1.28 (1.13-1.44)	<0.001	Yes (↑)
AKI	Magruder^33^ (THA)	2.80%	3.90%	OR, 0.69 (0.50-0.94)	0.0242	Yes (↓)
Sepsis	Verhey^39^	0.30%	0.30%	OR, 1.000 (0.488-2.048)	1	No
Sepsis	Magruder^34^ (TKA)	0.00%	0.40%	OR, 0.23 (0.09-0.48)	<0.001	Yes (↓)
Sepsis	Magruder^33^ (THA)	0%	0.40%	OR, 0.57 (0.17-1.42)	0.2821	No
Hypoglycemic event	Heo^37^	1.10%	0.90%	OR, 0.91 (0.42-1.97)	0.82	No
Hypoglycemic event	Magruder^33^ (THA)	0%	1%	OR, 0.45 (0.20-0.71)	0.0348	Yes (↓)
Hospital resource utilization						
Extended LOS (≥3 days)	Heo^37^	24.40%	28.50%	OR, 1.25 (1.05-1.49)	0.01	Yes (↓)
Average LOS	Magruder^34^ (TKA)	2.7 days	3.1 days	OR, 1.02 (0.95-1.10)	0.52	No
Average LOS	Magruder^33^ (THA)	2.7 days	2.9 days	OR, 0.99 (0.81-1.21)	0.9334	No
Average LOS	Kim^36^ (TKA)	2.7 days	2.9 days	Not specified	0.047	Yes (↓)
Average LOS	Kim^32^ (THA)	2.2 days	3.1 days	Not specified	0.001	Yes (↓)
Average same-day cost	Magruder^34^ (TKA)	$10,671.31	$11,484.50	Not specified	0.708	No
Average same-day cost	Magruder^33^ (THA)	$9,174.72	$10,046.30	Not specified	0.5169	No
Average 90-day cost	Magruder^34^ (TKA)	$15,291.66	$16,798.46	Not specified	0.012	Yes (↓)
Average 90-day cost	Magruder^33^ (THA)	$13,219.92	$14,681.71	Not specified	0.0562	Borderline (↓)

* SSI = surgical site infection, ED = emergency department, DVT = deep vein thrombosis, PE = pulmonary embolism, VTE = venous thromboembolism, CVA = cerebrovascular accident, MI = myocardial infarction, PNA = pneumonia, AKI = acute kidney injury, LOS = length of stay.

† The downward arrow indicates a significantly lower value in the GLP-1 group compared with the control group, and the upward arrow indicates a significantly higher value in the GLP-1 group.

Of the 4 studies reporting surgical site infection as an outcome^33,35,37,39^, none demonstrated significant differences between GLP-1 RA users and non-users. At the 2-year follow-up in the 3-cohort study on THA, the difference in component revision rates approached significance (p = 0.05), favoring GLP-1 RA users (<2.3%) over severely obese patients (3.9%) and morbidly obese non-users (3.1%)^32^.

### Medical Complications (Table 4)

The effects of GLP-1 RA on medical complications showed variable patterns. Magruder et al.^34^ reported higher rates of stroke (OR, 1.37; p = 0.01), deep vein thrombosis (OR, 1.50; p = 0.007), myocardial infarction (OR, 1.49; p = 0.003), pneumonia (OR, 1.67; p < 0.001), and acute kidney injury (OR, 1.28; p < 0.001) following TKA in GLP-1 RA users compared with controls. However, Magruder et al.^33^ showed contradictory results for acute kidney injury, reporting lower rates in the GLP-1 RA group (OR, 0.69; p = 0.0242) following THA. Magruder et al.^34^ demonstrated that GLP-1 RA users had significantly reduced odds of sepsis following TKA (OR, 0.23; p < 0.001), while Magruder et al.^33^ reported significantly reduced odds of hypoglycemic events following THA (OR, 0.45; p = 0.0348). In the 3-cohort analyses, Kim et al. found that GLP-1 RA users had significantly lower rates of any medical complication compared with severely obese patients and morbidly obese non-users in both TKA^36^ (10.6% versus 10.9% versus 12.7%; p = 0.014) and THA^32^ (10.5% versus 13.5% versus 14.1%; p = 0.03). GLP-1 RA users undergoing THA also had fewer hematomas (0% versus 1% versus 1.3%; p < 0.01)^32^.

### Hospital Resource Utilization (Table 4)

Hospital resource utilization outcomes generally favored GLP-1 RA users. Heo et al.^37^ reported significantly lower rates of an extended length of stay (≥3 days) among GLP-1 RA users compared with non-users (24.4% versus 28.5%), with non-users having higher odds of an extended stay (OR, 1.25; 95% CI, 1.05 to 1.49; p = 0.01). Similarly, Kim et al. demonstrated shorter hospital stays in both their TKA cohort^36^ (2.7 versus 2.7 versus 2.9 days for GLP-1 RA users, severely obese patients, and morbidly obese non-users, respectively; p = 0.047) and THA cohort^32^ (2.2 versus 2.7 versus 3.1 days, respectively; p = 0.001). In the 3-cohort analyses, GLP-1 RA users undergoing TKA had significantly lower 90-day readmission rates (5.3%) than severely obese patients (7.4%) and morbidly obese non-users (8.9%) (p < 0.001)^36^. Similarly, GLP-1 RA users undergoing THA showed lower 90-day readmission rates (6.9%) compared with severely obese patients (8.9%) and morbidly obese non-users (9.7%) (p = 0.04)^32^. These differences disappeared at the 2-year follow-up^36^. Magruder et al. found lower 90-day costs among GLP-1 RA users compared with controls in both THA^33^ ($13,219.92 versus $14,681.71; p = 0.0562) and TKA^34^ ($15,291.66 versus $16,798.46; p = 0.012).

GLP-1 RA use was associated with reduced hospital readmission rates in some studies, with Buddhiraju et al.^35^ and Magruder et al.^33,34^ reporting significant reductions ranging from 29% to 47%. Verhey et al.^39^ also found a significantly lower rate of outpatient visits in the GLP-1 RA group. However, Katzman et al.^38^ observed a higher rate of outpatient visits among GLP-1 RA users.

### Methodological Quality of the Studies

The studies demonstrated uniformly moderate overall quality of reporting, as shown in the Appendix.

## Discussion

Given the high prevalence of obesity among patients undergoing arthroplasty, GLP-1 RA use as a part of preoperative optimization protocols may reduce perioperative risks. However, the specific impact of GLP-1 RAs on hip and knee arthroplasty outcomes remains preliminarily studied, with definitive conclusions yet to be established. The present systematic review assessed the impact of GLP-1 RAs on total hip and knee arthroplasty outcomes by analyzing 8 retrospective studies comprising 22,611 GLP-1 RA users and 77,810 controls.

Hospital readmission rates showed the most consistently favorable results among GLP-1 RA users, with 3 studies^33-35^ reporting significant reductions associated with GLP-1 RA use, particularly during the 90-day postoperative period. Hospital resource utilization similarly favored GLP-1 RA therapy, with several studies documenting shorter hospital stays^32,36,37^ and lower 90-day costs^33,34^. PJI outcomes were promising but less consistent: while 5 studies demonstrated significant reductions associated with GLP-1 RA use^32-36^, 3 studies reported no significant differences^37-39^.

Revision surgery rates showed potential improvements favoring GLP-1 RA users in 3 studies^33,34,38^ but demonstrated no notable differences in 5 studies^32,35-37,39^. Medical complications yielded the most variable results, with some studies identifying increased vascular and pulmonary events among GLP-1 RA users and other studies observing reduced rates of sepsis and hypoglycemic events associated with GLP-1 RA use^33,34^.

Notably, even propensity score-matched studies yielded inconsistent results, suggesting genuine heterogeneity rather than study design limitations alone^33-35,38,39^. The 3-cohort design studies by Kim et al.^32,36^ demonstrated that GLP-1 RA users had superior 90-day outcomes compared with both morbidly obese non-users and severely obese patients. However, these early advantages diminished at the 2-year follow-up, suggesting that the benefits of GLP-1 RA may be limited to the immediate perioperative period rather than translating to long-term implant survival. This temporal pattern supports the proposed mechanisms of GLP-1 RA action, including improved glycemic control^40,41^, reduced systemic inflammation^42-44^, and improved wound healing^45,46^, indicating that the most effective role of GLP-1 RAs may lie in perioperative optimization rather than in altering long-term arthroplasty outcomes.

The heterogeneity in outcomes observed in the present systematic review likely stems from multiple sources. Patient characteristics vary considerably, with some individuals having elevated BMI but stable metabolic profiles, while others exhibit substantial metabolic dysfunction. These differences in inflammatory status and metabolic regulation may contribute to variations in perioperative risk and complication rates. Additionally, there exists wide variation in treatment protocols across clinical settings, including differences in dosing regimens, the timing of administration, and the duration of therapy. Individual GLP-1 RA agents also have distinct pharmacokinetic and pharmacodynamic properties, resulting in variable effects on weight loss, glycemic control, and inflammation. These sources of heterogeneity must be carefully considered when interpreting the current evidence and designing future research.

Our findings contrast with those of prior publications that have suggested more definitive benefits of GLP-1 RAs in arthroplasty outcomes. A recent meta-analysis reported significant reductions in PJI rates, concluding that GLP-1 RAs demonstrated perioperative benefits^23^. However, these apparently positive results were statistically fragile, losing significance when influential studies were removed in sensitivity analyses, and were based on a questionable quantitative pooling of methodologically heterogeneous studies. Similarly, a previous narrative review emphasized promising preclinical mechanisms and selective clinical findings while minimizing the substantial inconsistencies that were observed across human studies^24^.

The present systematic review has several important limitations. All included studies were retrospective, introducing inherent selection bias despite propensity score-matching efforts. The lack of randomized controlled trials limited causal inferences regarding the impact of GLP-1 RA on arthroplasty outcomes. Considerable heterogeneity was observed in treatment protocols, including in timing, duration, and the specific agents used, and there was incomplete reporting of dosage details. Inconsistent documentation of baseline BMI and glycemic control (e.g., glycated hemoglobin) further complicates the attribution of improved outcomes to weight loss versus metabolic effects. Most studies focused on short-term (90-day) outcomes, with limited long-term follow-up. These issues highlight the need for well-designed prospective trials with standardized protocols and extended follow-up.

Future research should determine whether the observed clinical benefits result from weight-mediated effects or direct pharmacological actions that are independent of BMI changes—a distinction that has crucial implications for clinical practice and patient selection criteria. Additional priorities include elucidating the mechanisms of periarticular soft-tissue effects, examining the durability of benefits beyond 2 years, and conducting appropriately powered randomized controlled trials that stratify outcomes by weight-loss response and have longer follow-up periods. Such studies could establish definitive clinical recommendations and potentially expand the therapeutic applications of GLP-1 RAs outside of metabolic disorders.

### Conclusions

Although GLP-1 RA therapy was associated with reduced hospital readmissions and decreased hospital costs within 90 days postoperatively in several studies, its benefits for PJI prevention showed mixed results in both TKA and THA, with some studies demonstrating a meaningful reduction in PJI and others showing no difference. No other clinical advantages were observed at the 2-year follow-up.

## Appendix

Supporting material provided by the authors is posted with the online version of this article as a data supplement at jbjs.org (http://links.lww.com/JBJS/J156).
